# Vegan, Vegetarian and Meat-Based Diets in Saudi Arabia

**DOI:** 10.7759/cureus.18073

**Published:** 2021-09-18

**Authors:** Noara AlHusseini, Muhammad Sajid, Yara Akkielah,, Touqa Khalil, Mayar Alatout, Peter Cahusac, Muhammad Faisal Ikram

**Affiliations:** 1 College of Medicine, Alfaisal University, Riyadh, SAU; 2 Pathology, Alfaisal University College of Medicine, Riyadh, SAU; 3 College of medicine, Alfaisal University, Riyadh, SAU; 4 Pharmacology & Biostatistics/Comparative Medicine, Alfaisal University College of Medicine, King Faisal Specialist Hospital & Research Centre, Riyadh, SAU; 5 Anatomical Sciences, Alfaisal University, College of Medicine, Riyadh, SAU

**Keywords:** saudi arabia, diet, omnivores, vegetarians, vegans, plant-based diets

## Abstract

Introduction

One of the most essential risk factors for chronic medical conditions is dietary intake. The dietary habits in Saudi Arabia shifted towards the Western diet, which is high in fat, salt and sugar. Plant-based diets like vegetarianism and veganism have gained popularity in the last few years. Individuals commit to a plant-based diet for many reasons. Plant-based diets are associated with various health benefits but can still cause nutrition deficiencies.

Purpose

The aim of the study is to examine the proportion of vegan, vegetarian and omnivore diets in Saudi Arabia. To compare between plant-eaters and meat-eaters in health, lifestyle factors and nutritional status.

Methods

A cross-sectional study was conducted. A previously validated online questionnaire was distributed via social media platforms. The authors used convenience sampling to collect the data.

Results

A total of 1018 respondents answered the survey. Most respondents 885 (87%) were omnivores, 52 were vegetarians (5%) and 81 (8%) were vegans. Moreover, 61% of the total respondents never consumed vitamin B12 supplements, and 10% consumed vitamin B12 daily. The majority of respondents 548 (54%) used no other dietary supplements, and 470 (46%) used unspecified dietary supplements. Vegan respondents were more likely than other diet categories to have healthier lifestyle features, including >3 times/week exercise (standardized residual = 3.55) and >7 hours of sleep (standardized residual = 2.44).

Conclusion

Majority of Saudis follow omnivore diets and the frequency of those who follow plant-based diets is very low. Those who follow a vegan diet seem to have better health rating and lifestyle compared to the omnivores. Public health officials and healthcare providers are encouraged to increase awareness among the Saudi population about the health benefits of a plant-based diet.

## Introduction

One of the most essential risk factors for chronic medical conditions is dietary intake. The dietary habits in Saudi Arabia shifted towards the Western diet, which is high in fat, salt and sugar. In 2013, poor diet accounted for 10.4% of disability-adjusted life years and 22.1% of deaths in Saudi Arabia [1]. Plant-based diets like vegetarianism and veganism have gained popularity in the last few years. A vegetarian diet excludes animal flesh, seafood, and their products from the diet [2]. Two types of vegetarian diets are lacto-vegetarianism, which incorporates dairy products in the diet, and ovo-vegetarianism, which incorporates eggs into the diet [2]. A vegan diet is a stricter type of vegetarianism, eliminating meat, seafood, and animal products, including but not limited to dairy, eggs, gelatine, whey, and honey [3]. Individuals commit to a plant-based diet for many reasons, including ethical motives, environmental considerations, concern with animal rights and animal welfare, social media influences, health benefits, and religious beliefs [4].

Scientific literature has shown that a plant-based diet is associated with various health benefits [5]. In particular, a plant-based diet may have positive effects on cardiovascular health and type 2 diabetes mellitus through modifiable factors such as body mass, serum glucose, blood pressure, and serum lipid profile [6-9]. In one study, the incidence and prevalence of diabetes were found to be the lowest among vegans [10]. Another noted effect of a plant-based diet is lower BMI and lower blood pressure [7]. Moreover, the risk of developing metabolic syndrome while following a plant-based diet appears to be reduced by half [9]. The low saturated fat and high unsaturated fat constituents of a balanced vegetarian diet may lower cardiovascular risk by improving blood lipid profile [9]. Therefore, meat-free dietary plans are not only suitable for preventing diseases but can also help in treating diseases [5]. In randomized controlled trials, a vegetarian diet appeared to have beneficial effects on the risk factors for cardiovascular diseases, including significant reductions in mean systolic blood pressure and on total cholesterol and LDL cholesterol levels [6]. However, it was also reported that these diets lowered the HDL cholesterol levels [6]. Both vegan and vegetarian diets are reported to be associated with a reduced risk of cancer [2].

Despite these favorable health outcomes related to plant-based diets, such restrictive dietary patterns tend to carry risks of nutritional deficiencies. Calcium, vitamin D, iron, zinc, and, most importantly, vitamin B-12 are commonly deficient nutrients that need to be supplemented regularly by individuals who follow a vegan or vegetarian diet [11]. On a larger scale, vitamin deficiencies play a role in the global burden of disease [12]. Micronutrient deficiencies may cause negative consequences such as impaired cognition, muscular pain, fatigue, weakness, neural tube defects, and increased risk of chronic disease risk [12].

Micronutrient deficiencies during pregnancy have been linked to lower rate of conception, length of gestation, and fetal development and growth, which can lead to pregnancy loss, preterm delivery, small birth size, birth defects, and fetal metabolic disturbances that can have effects extending well into adulthood [13]. More specifically, vitamins A, D, E, B12, and folate contribute toward protection against oxidative stress, boost immune cell proliferation, regulate epithelial integrity, and develop antigen action [12]. A recent review suggested that vitamin B12 deficiency increases the risk of tropical disease infections and adverse pregnancy outcomes as well as impaired psychomotor and cognitive development [12]. The main consequences of iron deficiency are anaemia, impaired cognitive and physical performance, increased maternal and child mortality, pallor, reduced physical endurance, and fatigue [14].

The accelerated urbanization of Saudi Arabia has led to many changes, most notably dietary changes. While traditional foods are still commonly consumed, Western dietary patterns, including vegetarianism and veganism, have started to appear in the food consumption patterns of the Saudi population [15].

The aim of this study is to examine the proportion of vegan, vegetarian, and omnivore diets in Saudi Arabia and to compare people who follow plant-based and omnivore diets in terms of health, lifestyle, and nutritional status. This study targets the adult population of Saudi Arabia, including natives and residents, on whom few studies in this area have been conducted. This will help us to better understand the dietary patterns of the Saudi Arabian population and gain insight into the healthy eating patterns in the region, which can complement the use of pharmaceutical medicine to prevent and treat non-communicable diseases.

## Materials and methods

This is a cross-sectional study utilizing an online questionnaire, which was distributed via social media platforms such as WhatsApp ©, Twitter ©, LinkedIn ™, and Facebook ™ from the 20th of September till the 10th of October 2020. The target population was adults 18 years and older residing in Saudi Arabia. Respondents under the age of 18 and anyone not residing in Saudi were instructed in the survey not to participate. The Study included both Saudis and non-Saudis who reside in Saudi Arabia. We used convenience sampling to recruit participants.

The questionnaire was adapted and modified from previously validated studies [16-19] to fit the aims of this study. The English version of the questionnaire was translated into the Arabic language by a linguistic expert, and face validity was achieved by a translation expert in the Arabic language. Content validity was achieved by a senior clinical dietitian at King Faisal Specialist Hospital. The first section of the survey includes demographic questions about age, gender, nationality, marital status, employment status, monthly income, and educational level. The second section covers questions related to the respondents’ diet, including type, duration, reasons for choosing a diet, and use of dietary supplements. In the third section, health-related questions cover respondents’ overall health, chronic medical conditions, height, and weight. The fourth section includes questions related to lifestyle, including exercise and sleep. The last section contains questions about the frequency of consumption of various food items. Food frequency questionnaires (FFQs) are used to measure long-term dietary intake; dietary information supplied by an FFQ is considered to be representative of a population’s dietary habits. FFQs are not designed to quantify the absolute intake of food items but rather to establish an individual’s ranking within a population with respect to intake. An FFQ comprises a list of food items and asks the respondents how often they consume each item. Items included in an FFQ are those that are often eaten by the population. Frequency categories must be mutually exclusive and have adequately narrow time intervals to capture the variability of consumption between respondents [20]​​​​​​. We adapted a FFQ from a previously validated survey and adjusted it to fit the Saudi diet; we received permission from the authors to adapt and use this questionnaire [13]. Approval from the Institutional Review Board at Alfaisal University has been granted IRB-20059.

Statistical analyses

Anonymized data in Excel data sheets obtained from the survey were transferred to Jamovi version 1.6. Frequencies were calculated for all variables. Descriptive analysis was performed for discrete and continuous variables, and Pearson’s correlations were performed.

Frequencies and correlations were also calculated for items in the FFQ. To reflect how much meat/red meat was in the respondents’ diets, we devised a weighted “carnivore score,” which was constructed from the following protein diet variables: Red meat (4 x score) + camel meat (3 x score) + liver (3 x score) + chicken (2 x score) + seafood (1 x score)+ eggs (1 x score) + falafel (1) + lentils (1). This gave a score that ranged from 1 to 58, the lower scores representing a vegan/vegetarian diet and the higher scores representing a carnivore diet. Relevant analyses involved Welch’s t tests, ANOVA, regression, and χ^2^. Statistical significance of 5% was used.

## Results

A total of 1,018 respondents completed the survey; their demographics are shown in Table 1.

**Table 1:** Demographic characteristics of the participants (N = 1,018).

		N	%
Age group	18-29 years	561	55%
	30-44 years	303	30%
	> 44 years	154	15%
Gender	Female	768	75%
	Male	250	25%
Marital status	Single	551	54%
	Married	447	44%
	Divorced/widowed	20	2%
Employment status	Yes	299	29%
	No	719	71%
Monthly income (Saudi Riyal)	<10,000 SR	179	23%
	10,000 to <20,000 SR	71	9%
	20,000+ SR	89	11%
	No income	449	57%
Level of education	High school, Diploma, or less	399	39%
	Bachelor’s degree	495	49%
	Postgraduate degree	124	12%
Nationality	Saudi	427	42%
	Non-Saudi	591	58%

The ratio of female to male participants was 3:1 (χ^2^(1) = 263.6, p < 0.001). Health, diet, and lifestyle information for the respondents is shown in Table 2.

**Table 2:** Health and lifestyle determinants of the participants (N = 1,018).

		N	%
Current diet type	Omnivores	885	87%
	Vegetarian	52	5%
	Vegans	81	8%
Vitamin B12 supplement intake frequency	Never	622	61%
	1-12 per year	133	13%
	2-5 per month	95	10%
	2-6 per week	63	6%
	Daily	105	10%
Using any other dietary supplements	Yes	470	46%
	No	548	54%
Chronic medical conditions	Yes	127	12%
	No	891	88%
Exercise frequency	Never	367	36%
	1-3 times per week	434	43%
	> 3 times per week	217	21%
Hours of sleep per night	0-3 hours	20	2%
	4-6 hours	508	50%
	>7 hours	490	48%
Length of being a vegetarian/vegan (in years)	< 1 year	25	18%
	1-2 years	26	19%
	2-5 years	46	34%
	> 5 years	40	29%
How much do the participants rate their health over the past 12 months	Very poor	9	1%
	Poor	46	4%
	Fair	140	14%
	Good	419	41%
	Very good	404	40%

**Figure 1:**
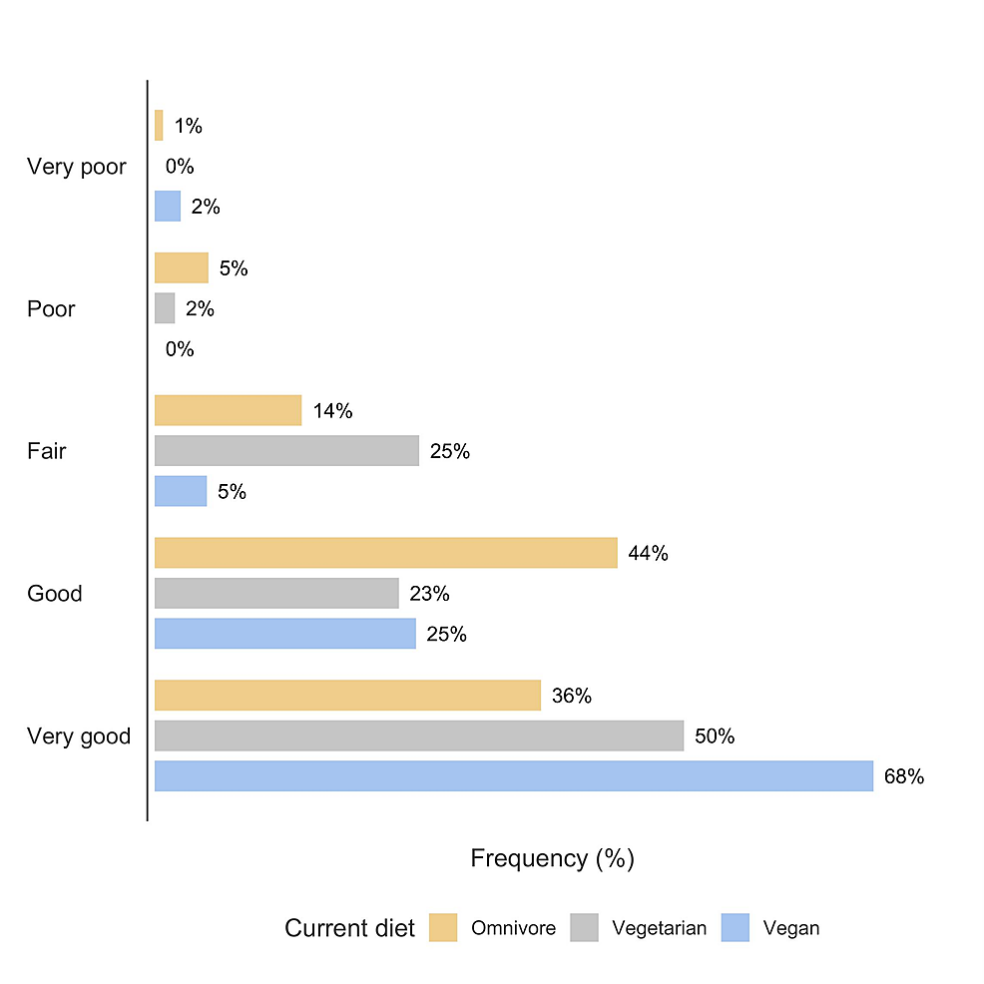
Reported health over the last 12 months according to current diet type.

Most respondents (885; 87%) were omnivores, while 52 were vegetarians (5%), and 81 (8%) were vegans. Moreover, 61% of the respondents never consumed vitamin B12 supplements, while 10% consumed vitamin B12 daily. The majority of respondents (548; 54%) used no other dietary supplement, while 470 (46%) used unspecified dietary supplements. A total of 127 respondents (12%) had one or more chronic medical condition, and only 55 (5%) rated their health over the past 12 months as poor or very poor. There was no obvious association between current diet type and chronic health conditions (χ^2^(2) = 3.79, p = 0.151). A 3 × 5 contingency table analyses showed a strong association between current diet type and self-rated health over the past 12 months (χ^2^(8) = 48.08, p < 0.00001), with vegans more likely than other diet categories to report very good health (standardized residual = 4.03) (see Figure 1).

The majority of respondents (651; 64%) engaged in regular exercise, while 36% reported that they never exercise. Nearly half of the sample (48%) had >7 hours of sleep per night, 49% got 4-6 hours of sleep, and 2% (20) got 0-3 hours of sleep. A 3 × 3 contingency table analysis found associations between current diet type and frequency of exercise (χ^2^(4) = 19.17, p = 0.0007) and between current diet type and hours of sleep (χ^2^(4) = 13.62, p = .009). In each of these analyses, vegan respondents were more likely than other diet categories to have healthier lifestyle features, including >3 times/week exercise (standardized residual = 3.55) and >7 hours of sleep (standardized residual = 2.44).

These analyses indicate that the vegan diet is associated with healthier lifestyle features. To quantify the association more precisely, we used a carnivore score (as described in the Statistical Analyses section) as a predictor rather than the current diet category, on the assumption that the lower the intake of meat (in particular, red meat), the healthier the respondent would be. Other variables were also included in a regression model, with the reported 12-month health as the outcome variable.

A cross-correlation matrix for all of the measurement variables was obtained. Since the sample included more females than males, we looked at how other variables differed according to sex. The male carnivore score of 32.5 was higher than the score of 29.0 for females (t(401) = 7.09, p < 0.00001), and the females were older than the males (χ^2^(2) = 21.63, p < 0.001). The females were twice as likely as the males (odds ratio = 2.045) to be unemployed (χ^2^(1) = 22.35, p < 0.001) and had less income than the males (χ^2^(3) = 25.92, p < 0.001) but tended to be better educated than males (χ^2^(2) = 8.66, p = 0.013) (although this was age-dependent). The females were less likely than males to exercise regularly (χ^2^(2) = 6.55, p = 0.038). These differences indicated that there was a heterogeneity of subsamples for sex and that sex should be included as a factor.

Plausible nominal variables were selected for multiple regression. The initial model included sex, BMI, chronic medical condition, sleep, exercise, age, employment, income, carnivore score, nationality, vitamin B frequency, and education. After removing the variables that explained the least of the variance, the following variables remained: chronic medical condition, BMI, sleep, exercise, age, sex, employment, and carnivore score. Because of the high female to male ratio and because males were twice as likely to be employed as females, an interaction term between sex and employment was entered into the model. The final model explained 10.2% of the variance (R²=0.10196) and (P-value < .00001). Although this represented a moderate amount of the total variance, many of the explanatory variables had relatively strong standardized estimate values that were statistically significant. 

The strongest predictors for how healthy respondents felt over the last 12 months were chronic medical condition (reported health decreased when present), employment (reported health increased when present), sex (reported health decreased for females), and the interaction between sex and employment. The weakest predictors were BMI and carnivore score; as these increased, respondents reported feeling less healthy. All predictors in the model, except for the sex x employment interaction, achieved p < 0.05. Assumption checks included Q-Q and residual plots, which were satisfactory. The collinearity statistics were acceptable, the largest being for the interaction between sex and employment at 6.0, but within recommendations Wetherill et al., 1986. Table 3 provides a summary of the regression, the regression model coefficients, and the associated statistics. Overall Frequencies and percentages of reported Food frequency questionnaire are presented in Table 4. 

**Table 3:** Model coefficients for multiple regression-dependent variable: health over last 12 months.

Predictor	Estimate	SE	t	p	Stand. Estimate
Intercept	3.53583	0.25501	13.86528		
Hours sleep	0.21837	0.05007	4.36153	0.00001	0.13294
How often exercise	0.20135	0.03615	5.56958		0.16966
Carnivore score	-0.00784	0.00410	-1.91090	0.05630	-0.06011
Age	0.14925	0.04254	3.50832	0.00047	0.12461
BMI	-0.01228	0.00468	-2.62697	0.00875	-0.08442
Chronic medical:					
No – Yes	0.33034	0.08533	3.87146	0.00012	0.37434
Employment:					
No – Yes	-0.33435	0.11355	-2.94446	0.00331	-0.37889
Sex:					
Female – Male	-0.25427	0.10492	-2.42359	0.01555	-0.28814
Sex ✻ Employment:					
(Female – Male) ✻ (No – Yes)	0.25359	0.13090	1.93725	0.05300	0.28736

**Table 4:** Overall frequencies and percentages of reported food frequency questionnaire (FFQ).

Food Categories according to FFQs	Never in the last year	1-2 times a month	1-3 times a week	Daily
	n (%)	n (%)	n (%)	n (%)
Protein [Meat (Mutton, beef, shawarma, stew)]	754 (64.1)	383 (32.5)	0 (0)	39 (3.3)
Protein [Chicken (grilled, fried, stew, kebab, shawarma)]	122 (10.3)	463 (39.3)	409 (34.7)	183 (15.5)
Protein [Sea food (fish, tuna, shrimp, prawns, crab)	85 (7.2)	1080 (92.1)	0 (0)	6 (0.5)
Protein [Falafel]	140 (11.9)	733 (62.7)	254 (21.7)	42 (3.5)
Protein [Eggs]	68 (5.8)	411 (35.0)	413 (35.2)	280 (23.8)
Carbohydrates [Rice]	27 (2.2)	390 (33.1)	232 (19.7)	527 (44.8)
Carbohydrates [Bread]	46 (3.9)	640 (54.6)	220 (18.7)	265 (22.6)
Carbohydrates [Pies (pizza/samosa)]	60 (5.1)	776 (65.9)	305 (25.9)	35 (2.976)
Carbohydrates [Pasta]	256 (21.8)	715 (61.1)	144 (12.3)	54 (4.6)
Carbohydrates [Potatoes, pastries]	11 (0.9)	438 (37.2)	694 (58.9)	34 (2.8)
Carbohydrates [Cereals, pudding]	62 (5.2)	595 (50.2)	330 (27.8)	197 (16.6)
Dairy and Fat [Cheese]	121 (10.2)	500 (42.5)	327 (27.8)	227 (19.3)
Dairy and Fat [Yoghurt]	303 (25.9)	678 (58.0)	120 (10.2)	67 (5.7)
Dairy and Fat [Cream]	284 (24.3)	540 (46.2)	262 (22.4)	82 (7.0)
Dairy and Fat [Laban, milk]	267 (22.9)	436 (37.5)	227 (19.5)	232 (19.9)
Dairy and Fat [Mayonnaise]	299 (25.5)	517 (44.1)	256 (21.8)	98 (8.3)
Snacks [Ice cream]	411 (34.9)	643 (54.6)	84 (7.1)	38 (3.2)
Snacks [Chocolate]	118 (10.0)	827 (70.3)	161 (13.6)	70 (5.9)
Snacks [Potato chips]	93 (7.9)	638 (54.2)	314 (26.7)	131 (11.1)
Snacks [Nuts]	126 (10.6)	706 (59.7)	231 (19.5)	118 (9.9)
Snacks [Biscuits]	95 (8.1)	677 (57.6)	284 (24.1)	118 (10.0)
Snacks [Cakes/ dessert]	125 (10.6)	772 (65.6)	217 (18.4)	62 (5.2)
Snacks [Candy]	291 (24.8)	747 (63.8)	105 (8.97)	27 (2.3)
Snacks [Sugary drinks]	190 (16.1)	709 (60.3)	180 (15.3)	96 (8.1)

The food most commonly reported to be consumed by the respondents was chicken with almost 50% of the population either consuming it daily or one to three times per week. This was followed by rice, which was consumed by 44% of the respondents daily, and bread, consumed daily by 40% of the respondents. Among dairy products, milk and laban (white thick liquid derived from yogurt) were reported to be the most frequently consumed. Potato chips was the most frequently reported snack, consumed by 40% of the population more than three times per week. A Pearson’s correlation between the most frequently consumed foods and BMI is shown in Table 5. 

**Table 5:** Pearson’s correlation between BMI and most frequently consumed foods.

	BMI	
Food item	Pearson's r	p-value
Protein [Chicken]	0.136	0.0000
Carbohydrates [Rice]	0.009	0.7798
Carnivore score	0.173
Dairy and Fat [Milk]	0.061	0.0549
Dairy and Fat [Laban]	0.027	0.3908
Snacks [Potato crisps ]	-0.112	0.0004

## Discussion

Our results revealed that the majority of our respondents (885; 87%) were omnivores, and the frequency of respondents reporting vegetarian and vegan diets was low (5% and 8%, respectively). This is the first study conducted in Saudi Arabia that has reported the frequency of vegetarian and vegan diets in the general population. The main reason respondents’ reported for adopting a vegetarian/vegan lifestyle was health, and this is in agreement with previous studies [21].

A nationally representative study of Saudis (n = 10,735) revealed that only 5% of the Saudi population met the dietary guideline recommendations for fruits, and only 7.5% met the recommendations for vegetables [22]. Another nationwide, cross-sectional study (n = 3,699) found that the consumption by Saudis of fruits and vegetables was only 5% and 7.3% of the recommended amount, respectively, which is well below the nutritional requirements [23]. Plant-based diets are reported to be low in energy density, saturated fat, and sodium, which can positively affect cardiovascular health [6]. Plant-based diets are also high in fiber and unsaturated fats, and this can also have a positive effect on cardiovascular health [6]. Additionally, replacing animal-based foods, especially red meat, with a plant-based diet can help in lowering the levels of sodium, nitrate, and nitrites, which have been shown to be linked to poor cardiovascular health [6,8]. The results were expected since the dietary habits in Saudi lack sufficient intake of fruits and vegetables, hence plant-based diets are not very common in the country. 

In our study, 61% of the respondents never consumed vitamin B12 supplements, while 10% consumed vitamin B12 daily. The majority of respondents (548; 54%) used no other dietary supplement, and 470 (46%) used unspecified dietary supplements. These results align with another local study, which confirmed that 44.6% of the Saudi population used dietary supplements and multivitamins; the most commonly used were vitamins C and D, iron, and calcium [24]. Regarding vitamin B12 use, many respondents might not be aware that certain dietary supplements such as multivitamins usually include B12. The high intake of dietary supplements can be due to the population's knowledge of their poor dietary habits and their willingness to augment or improve nutritional deficiencies. 

Our results showed that vegans were more likely than other diet categories to report good health and healthy lifestyle features, including >3 times/week exercise and >7 hours of sleep. Many studies support our findings in terms of the health benefits of vegan diets. Glick-Bauer et al. (2014) examined the impact of diets on gut microbiota; they concluded that vegan guts showed a reduced abundance of pathobionts and a greater abundance of protective species that reduce the level of gut inflammation [25]. Another study also confirms our findings as it revealed that individuals following a plant-based diet exhibited lower blood pressure compared with individuals following an omnivorous diet, independently of salt intake, body weight, and activity levels [7]. In a meta-analysis of randomized controlled trials, a plant-based diet reduced the total HDL cholesterol ratio and, subsequently, the risk of cardiovascular disease in a dose-response manner, which was further corroborated in prospective cohort studies [9]; these results align with our results as, in our study, the vegan/vegetarian respondents reported very good health. Many studies have reported that the health benefits associated with vegan and vegetarian diets are an important method to improve health and well-being and prevent chronic, life-style associated diseases [9].

The strongest predictor for how healthy respondents felt over the last 12 months was the employment status (decreased health for unemployed). The weakest predictors were BMI and carnivore score; as these increased, respondents reported feeling less healthy. A study that explored the effect of a plant-based diet, especially the high fibre content associated with it, revealed that a plant-based diet can be healthy and nutritious and can reduce the risk of chronic diseases such as type 2 diabetes, cancer, and cardiovascular diseases. An omnivore diet leads to the consumption of unhealthy fats and, thus, can have a negative effect on health [26]. A randomized controlled trial in New Zealand (controls n = 32, intervention n = 33) found that following a plant-based diet reduced BMI and cholesterol levels [27].

In our study, the data from the respondents’ FFQs showed a high frequency of consumption of chicken on a daily basis or more than one to three times per week. The protein (chicken) and carnivore scores showed a good correlation with BMI, but for potato chips, the correlation was negative. We hypothesize that overweight respondents might try to avoid eating potato chips, leading to this correlation. Our results are in concordance with other local studies that show limited fruits and vegetables intake but instead higher intake of protein and carbohydrates [22,23]. It is highly encouraged to emphasize the positive impact of plant-based diets among the Saudi population in terms of controlling obesity and preventing cardiac diseases [28]. These are unique and new findings not previously reported from Saudi Arabia in the published literature. The study has a cross-sectional design, which prevents any causal inference. Another limitation could be recall bias in the respondents’ reports of their food intake and weight. However, this study is unique because there are limited studies that show the dietary habits of the Saudi population and the lack of plant-based food consumption. Future studies are essential to better understand the frequency of plant-based diets in the Saudi population and their associated benefits and risks. Public health officials and healthcare providers are encouraged to increase awareness among the Saudi population about the health benefits of a plant-based diet in preventing obesity and cardiac diseases. 

## Conclusions

The majority of the respondents in our study follow an omnivore diet, and the frequency of those who follow a plant-based diet is very low. Those who follow a vegan or vegetarian diet had a better self-rated health and healthier lifestyle compared to the omnivores. A plant-based diet can be customized to fit Saudi cultural preferences and, with large-scale adoption, could mitigate the health and environmental threats associated with an animal-based diet.
